# Greeting—New Year

**Published:** 1891-12

**Authors:** 


### GREETING.

THE JOURNAL extends greeting to its many readers, and thanking them for the aid, encouragement and support so generously accorded us in our efforts to maintain the dignity of legitimate medicine, and to reflect the better aspect of Texas medicine,—wishes them all a merry Christmas and a happy New Year! To its advertising patrons the JOURNAL extends "the compliments of the season" and wishes them a world of prosperity and success.

The JOURNAL, thank you, is doing pretty well. Subscriptions are still in order; so are renewals. Do not forget the little RED-BACK in your rejoicing; $2 makes a very neat Christmas gift.

